# Metformin in aging and aging-related diseases: clinical applications and relevant mechanisms

**DOI:** 10.7150/thno.71360

**Published:** 2022-03-06

**Authors:** Sheng Chen, Donghao Gan, Sixiong Lin, Yiming Zhong, Mingjue Chen, Xuenong Zou, Zengwu Shao, Guozhi Xiao

**Affiliations:** 1 Department of Orthopaedics, Union Hospital, Tongji Medical College, Huazhong University of Science and Technology, Wuhan 430022, China.; 2 Department of Biochemistry, School of Medicine, Southern University of Science and Technology, Guangdong Provincial Key Laboratory of Cell Microenvironment and Disease Research, Shenzhen Key Laboratory of Cell Microenvironment, Shenzhen, 518055, China.; 3 Guangdong Provincial Key Laboratory of Orthopaedics and Traumatology, Department of Spine Surgery, The First Affiliated Hospital of Sun Yat-sen University, Guangzhou, 510080, China.

**Keywords:** Metformin, aging, aging-related diseases, clinical application, molecular mechanism.

## Abstract

Aging is a natural process, which plays a critical role in the pathogenesis of a variety of diseases, i.e., aging-related diseases, such as diabetes, osteoarthritis, Alzheimer disease, cardiovascular diseases, cancers, obesity and other metabolic abnormalities. Metformin, the most widely used antidiabetic drug, has been reported to delay aging and display protective effect on attenuating progression of various aging-related diseases by impacting key hallmark events of aging, including dysregulated nutrient sensing, loss of proteostasis, mitochondrial dysfunction, altered intercellular communication, telomere attrition, genomic instability, epigenetic alterations, stem cell exhaustion and cellular senescence. In this review, we provide updated information and knowledge on applications of metformin in prevention and treatment of aging and aging-related diseases. We focus our discussions on the roles and underlying mechanisms of metformin in modulating aging and treating aging-related diseases.

## 1. Introduction

Aging is broadly defined as an irreversible and inevitable biological process, which is characterized by progressive deterioration of the social, physical and mental conditions of the individual organism with advancing chronological age, which generally starts after sexual maturity and ultimately results in morbidity 1. From the mid-1900s, researchers have been debating whether aging is the cause of aging-related diseases, including diabetes mellitus, degenerative musculoskeletal diseases, cardiovascular diseases, neurodegenerative diseases, cancers and other diseases 2. It is now widely believed that aging is one of the main risk factors in the occurrence of these diseases 3.

Nowadays, as the average human life expectancy and the proportion of older people increase, the global financial burden of aging-related diseases becomes increasingly heavy, and effective preventive approaches and therapeutic methods are urgently needed. To comprehensively dissect the various factors in the aging process and identify relevant intervention targets, Lopez-Otin et al. 4 proposed nine tentative hallmarks, which are considered to contribute to the initiation and progression of aging and together determine the phenotypes of aging. The nine aging hallmarks include four primary hallmarks (loss of proteostasis, telomere attrition, genomic instability and epigenetic alterations), three antagonistic hallmarks (dysregulated nutrient sensing, mitochondrial dysfunction and cellular senescence) and two integrative hallmarks (altered intercellular communication and stem cell exhaustion). Better understanding of interplays among these hallmarks and their respective contributions to the aging process will largely help identify pharmacological targets for aging and aging-related diseases.

Metformin (N, N-dimethylbiguanide), which contains two linked guanidine rings and belongs to the biguanide class of hypoglycemic drugs, was originally derived from a medicinal herb French lilac Galega officinalis 5. It was first described in Culpeper's Complete Herbal in 1653 that Galega officinalis could be used to treat worms, fever, pestilence, epilepsy and other ailments 6. In 1772, Galega officinalis was recommended by John Hill to treat symptoms of diabetes 6. In 1957, French physician Jean Sterne pursued this antihyperglycemic property and first reported the application of metformin in the treatment of diabetes 6. Since then, metformin has been widely used in Europe to treat diabetes. But it was slow for metformin to get the approval in the United States because of the concern over lactic acidosis. In 1995, metformin was proven effective and safe in the treatment of diabetes, and introduced in US. At present, metformin has become the first-line medicine to treat type 2 diabetes (T2D). Recently, numerous studies using different cell lines and model organisms have demonstrated that metformin has a great potential to retard aging and alleviate aging-related diseases by targeting key molecules related to aging 7. Moreover, a multicenter, randomized, double-blind, placebo-controlled clinical trial Targeting Aging with Metformin (TAME) has been initiated to further explore the anti-aging role of metformin 8.

In this review, we provide up-to-date information and knowledge on the clinical applications of metformin in the prevention and treatment of major aging-related diseases. We review and discuss the roles and underlying mechanisms of metformin in modulation of aging hallmarks and treatment of aging-related diseases.

## 2. Pharmacology of metformin

### 2.1 Chemistry

**Figure 1:**
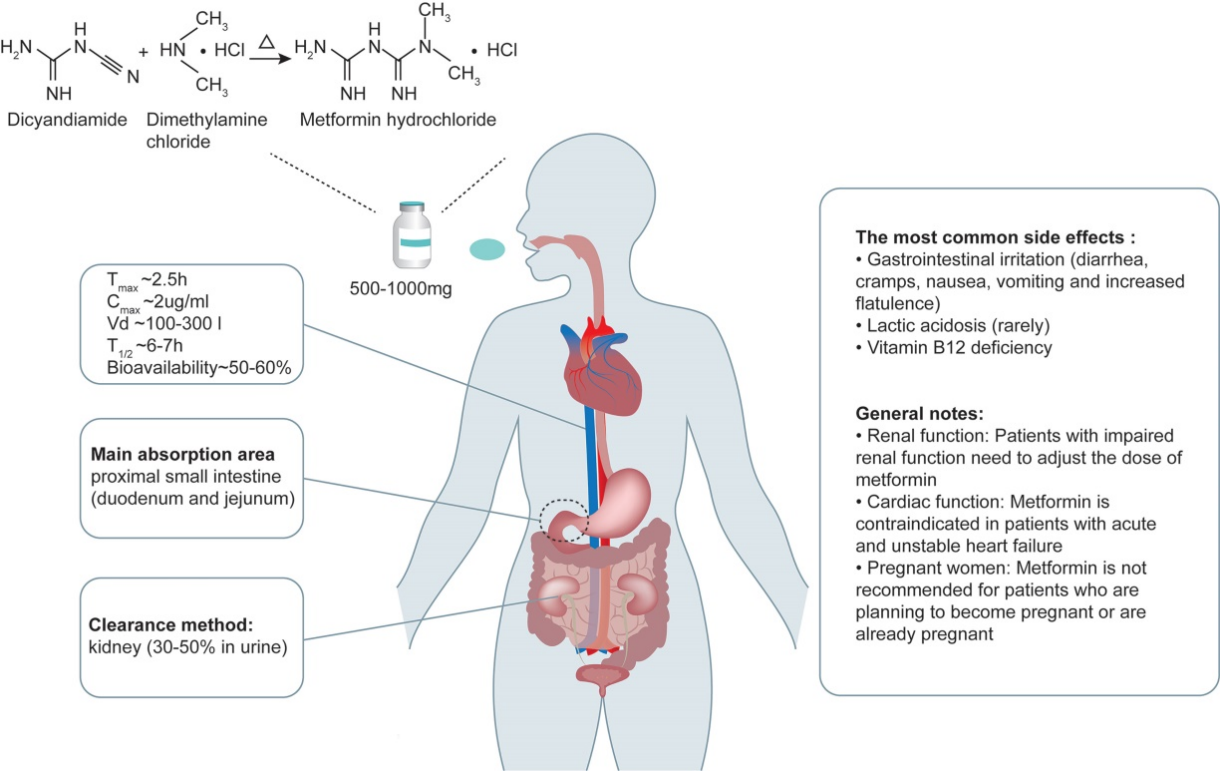
** Metformin chemistry, pharmacokinetics and side effects.** Metformin contains a double salt group and is synthesized from dimethylamine chloride and dicyandiamide. The oral dose of metformin is 500-1000mg, and the absolute bioavailability of oral metformin hydrochloride is relatively low (about 50-60%), and its absorption process is mainly in the proximal intestine, including the duodenum and jejunum. The T_max_ is about 2.5 hours. Its typical peak plasma concentration (C_max_) is about 2 μg/ml, and rarely exceeds 4 μg/ml. And the state concentration range is 0.3-1.5 μg/ml. The absorption of metformin in the gastrointestinal tract (GIT) is slow and incomplete. Metformin is not metabolized by the liver. Plasma protein binding is negligible and widely distributed (usual volume of distribution [V_d_], 100-300l). Metformin has an elimination half-life (T_1/2_) of ~6-7h. The main way of elimination is rapid excretion through the kidneys, where 30-50% of the metformin is eliminated and remains unchanged in the urine. The most common side effects include gastrointestinal irritation, lactic acidosis and vitamin B12 deficiency. Adapted from Bailey et al. 6.

The structural formula of metformin is C_4_H_11_N_5_ and its molecular weight is 129.16 g/mol, consisting of planar molecule with single protonation between two imino groups and two non-polar methyl groups. The metformin used in clinic (metformin hydrochloride) is synthesized from chlorinated dimethylamine and dicyandiamide (Figure 1), rather than from plants. The molecular formula of metformin hydrochloride is C_4_H_11_N_5_•HCL and its molecular weight is 165.6 g/mol. Metformin hydrochloride is a white powder at room temperature, with a melting point of 223-226 ^o^C, and its solubility in water can be as high as 200 g/L.

### 2.2 Pharmacokinetics

After oral administration, the absorption process of metformin is completed mainly in the proximal small intestine (duodenum and jejunum). In healthy people, regardless of the dose of metformin, the absorption process is completed within 6-10 hours after oral administration of metformin. And the absolute bioavailability of oral metformin hydrochloride is relatively low (about 50-60%) 9. Eating reduces the degree of absorption of the drug and slightly delays its rate of absorption. After oral administration, the accumulation of metformin can be detected in salivary glands, intestinal cells, red blood cells, esophagus and kidneys, and 20-30% of metformin can be recovered in feces. Of note, metformin is not metabolized by the liver. The main way of elimination of metformin is through rapid excretion by the kidneys, where 30-50% of the metformin dose is eliminated and remains unchanged in the urine 10 (Figure 1). Therefore, the dose of metformin needs to be adjusted in patients with impaired renal function. Metformin seems to have no short-term adverse effects on pregnancy, but its long-term safety is unclear 11 . Thus, the decision to use metformin during pregnancy should be based on assessing the risks of each mother and baby, and it is not recommended for patients who are planning to become pregnant or are already pregnant to use metformin. Metformin can be used alone or in combination with other drugs. Proton pump inhibitors were reported to reduce the transport of metformin by inhibiting organic cation transporter (OCT) 1, OCT2 and OCT3 12; however, its implication in human body is unclear.

### 2.3 Side effects

The most common side effect of metformin is its gastrointestinal irritation, which causes flatulence, diarrhea, nausea, vomiting, and cramps. These symptoms are most common when metformin is used for the first time or when the dose is high. This discomfort can be usually avoided by starting with a low dose and gradually increasing the dose or using sustained-release formulations. The most serious adverse effect of metformin is lactic acidosis. This complication is rare and the vast majority of cases seem to be related to liver and kidney damage, rather than metformin itself 13. In addition, it has been reported that high-dose and prolonged use of metformin increases the incidence of vitamin B12 deficiency 14. Therefore, it is helpful to screen patients taking metformin for vitamin B12 deficiency. Although high-dose administration of metformin can cause a series of side effects, results from studies in cultured cells treated with high concentration of metformin are interesting. For example, Virtanen et al 15 showed that 600 μg/mL metformin significantly inhibited angiogenesis, while 5 or 50 μg/mL metformin had no effect. In line with it, Dallaglio et al 16 revealed that 10 mM (about 1290 μg/mL) metformin, which greatly exceeds the concentration *in vivo*, had an antiangiogenic effect in endothelial and tumor cells. These results suggest that high-dose administration of metformin may be beneficial for antiangiogenic therapy for diseases, such as cancer.

## 3. Clinical applications

In addition to diabetes mellitus, metformin has been proved to be effective for aging-related diseases, such as degenerative skeletal diseases, cardiovascular diseases, neurodegenerative diseases, tumors, obesity and other metabolic abnormalities (Table 1).

### 3.1 Aging and lifespan

Since 2015, the World Health Organization has officially recognized aging as a disease, which greatly stimulates the research on aging and aging-related diseases as well as the development of relevant therapeutic strategies. Several epidemiological studies demonstrated that metformin reduced the incidence of multiple age-related diseases as well as all-cause mortality. Importantly, this phenomenon was observed not only in diabetic patients but also in non-diabetic patients 17, 18. Result from a randomized, double-blind, placebo-controlled, crossover trial revealed that metformin had both metabolic and non-metabolic effects associated with aging in the elderly, providing evidence for the anti-aging effect of metformin 19. This notion is further supported from results from preclinical studies showing that metformin could extend the lifespan of C. elegans, an invertebrate, and mice 20, 21.

### 3.2 Diabetes mellitus

The prevalence of diabetes, especially T2D, increases with the age. Furthermore, individuals with diabetes are more likely to develop other age-related comorbidities, such as mild cognitive impairment, Alzheimer's disease, cardiovascular disease, osteoporosis, visual impairment, and renal dysfunction, suggesting that diabetes itself may represent a pro-aging state 22. As the most widely used T2D drug in the world, metformin has been used in clinical practice for more than 50 years. A large number of studies and clinical trials demonstrate that metformin alone or in combination with other hypoglycemic drugs is effective in the treatment of T2D. In 1991, Hermann and coworkers reported that metformin restored the fasting blood glucose (FBG) of patients with non-insulin-dependent diabetes mellitus (NIDDM) 23. In a 1995 study by Ra DeFronzo, metformin monotherapy was shown to improve blood glucose control and lipid concentration in patients with NIDDM 24. The same group further reported that metformin reduced fasting blood glucose and glycosylated hemoglobin in a dose-dependent manner. In recent years, cumulative evidence suggests that the effect of combination of metformin with other drugs is better than that of metformin used alone. Drugs that are used in combination with metformin include glibenclamide, troglitazone, insulin, dipeptidyl peptidase 4 (DPP4) inhibitors, sodium-dependent glucose transporters 2 (SGLT2) inhibitors and Glucagon-like peptide 1 (GLP1) receptor agonists. For example, the combination of metformin and glibenclamide displayed a better effect on reducing the level of blood glucose than that when it was used alone 25. Similarly, the combined use of metformin and troglitazone reduced the production of endogenous glucose while increasing the excretion of peripheral glucose, thereby better controlling the blood sugar level of T2D patients 26. Furthermore, adding metformin to insulin therapy was reported to better control the blood sugar level 27.

### 3.3 Degenerative skeletal diseases

**Figure 2:**
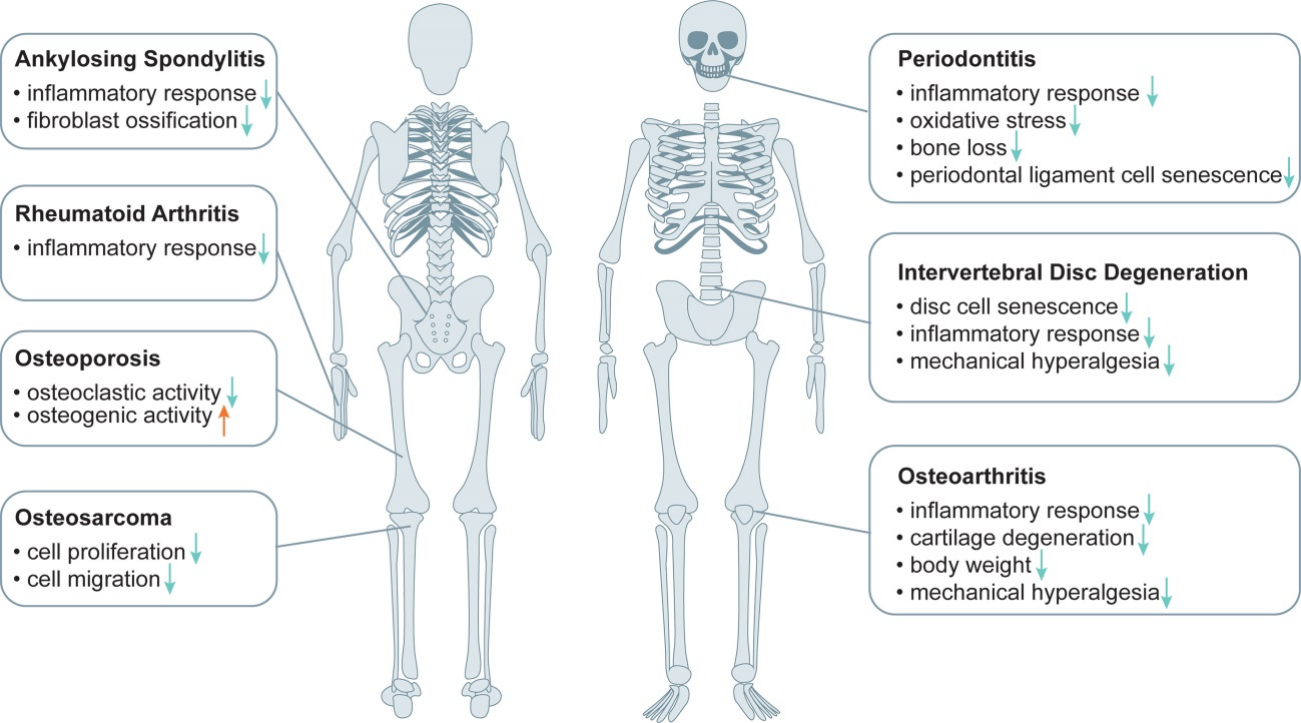
** The effect of metformin in the treatment of musculoskeletal diseases.** Metformin plays an important role in the treatment of musculoskeletal diseases such as osteoarthritis (OA), osteoporosis (OP), intervertebral disc degeneration (IVDD), periodontitis (PD), ankylosing spondylitis (AS), rheumatoid arthritis (RA), osteosarcoma (OS) by inhibiting the effect of inflammatory response, cartilage degeneration, mechanical hyperalgesia, cellular senescence, osteoclastic activity, oxidative stress, fibroblast ossification, cellular proliferation and migration, reducing body weight or improving osteogenic activity.

Osteoporosis (OP), osteoarthritis (OA) and intervertebral disc degeneration (IVDD) are major degenerative skeletal diseases, which are all related to aging. Increasing evidence suggests that metformin plays a beneficial role in treatment of degenerative skeletal diseases (Figure 2).

OP is an important metabolic bone disease, which is associated with diabetes. Clinical cohort studies showed that metformin treatment was associated with a lower rate of OP and a lower risk of fracture in diabetic patients compared with those who did not receive metformin treatment 28, 29. Result from a phase II clinical study showed that metformin improved bone metabolism caused by glucocorticoid overexposure through inhibition of bone resorption and stimulation of bone formation in trabecular bone 30.

OA is the main cause of severe joint pain, physical disability and impaired quality of life in the aging population. Age-related cell senescence and increased expression of inflammatory mediators are two important contributors to OA development and progression. The body weight reducing effect of metformin might indirectly improve the effect of obesity on OA 31. Metformin displayed a beneficial effect on long-term knee outcomes in obese patients with knee joint OA 32. Moreover, preclinical evidence shows that metformin plays a role in the treatment of OA due to its anti-inflammatory and skeletal regulatory effects 33, 34. Animal studies showed that metformin protected articular cartilage by activating AMPK pathway, delayed the onset and progression of OA and reduced OA-related pain sensitivity in injury-induced OA models in mice and in primates 35, 36.

Intervertebral disc (IVD) is composed of the cartilage endplate, an outer annulus fibrosus and a central nucleus pulposus, and its degeneration, namely IVDD, leads to vertebral instability and neurological symptoms 37, 38. While clinical evidence is still lacking, results from cultured cells and preclinical studies suggest that metformin plays a beneficial role in treatment of IVDD. Metformin was shown to improve the model of punctation-induced disc degeneration in rats and reduce local mechanical hyperalgesia, possibly by inhibiting cellular senescence and inflammatory responses in the nucleus pulposus and annulus fibrosus 39, 40. Liao et al. showed that extracellular vesicles derived from metformin-treated bone marrow mesenchymal stem cells improved disc cell senescence both *in vitro* and *in vivo* 41.

In addition to OP, OA and IVDD, preclinical studies indicate that metformin plays a beneficial role in the treatment of other degenerative skeletal diseases, such as rheumatoid arthritis (RA) and periodontitis (PD). Results from several studies reveal that aging is an important risk factor for the progression of RA, which is closely associated with inflammatory activity 42. The anti-inflammation effect of metformin can contribute to its treatment of RA 43. PD is a chronic inflammatory disease involving periodontal tissue, and its incidence is increasing with the aging of the population. Metformin was shown to reduce the inflammatory response, oxidative stress and bone loss in rats with PD, and alleviate oxidative stress-induced aging by stimulating autophagy, which may benefit PD treatment from the perspective of anti-aging 44, 45. Additionally, *in vitro* studies showed that metformin effectively inhibited the ossification and inflammation of ankylosing spondylitis (AS) fibroblasts by activating PI3K/AKT and AMPK pathways, suggesting that metformin may be developed as a potential drug for the treatment of AS 46. And metformin was found to inhibit osteosarcoma (OS) cell proliferation and migration by affecting AKT activity, and may reduce the cancer risk of continuous use of metformin in T2D patients 47, 48. Collectively, metformin may be used as a potential drug for the treatment of degenerative skeletal diseases and other bone disease.

### 3.4 Cardiovascular diseases

Aging-related cardiovascular diseases include a variety of categories, such as coronary heart disease and heart failure. Growing evidence from a number of studies suggest that metformin can protect against cardiovascular diseases. Results from several clinical trials showed that metformin mitigated the development of cardiovascular disease and reduced morbidity in diabetic patients 49, 50. It is important to investigate the cardiovascular effects of metformin in non-diabetic patients because high blood sugar itself is a significant factor affecting vascular environment. Current clinical studies on the cardioprotective effects of metformin have primarily been focused on coronary heart disease, heart failure, and heart attack combined with pulmonary hypertension. Sardu et al. reported that metformin reduced the risk of coronary heart disease by reducing coronary endothelial dysfunction 51. Moreover, metformin attenuated the early progression of coronary plaque in men with prediabetes 49. Zhang et al. reported that metformin changed the composition of serum lipids and thereby indirectly reduced the probability of cardiovascular events in patients 52. Furthermore, metformin protected the heart by reducing myocardial oxygen consumption and significantly reduced left ventricular mass index (LVMI), left ventricular mass (LVM), office systolic blood pressure and oxidative stress in patients with coronary artery disease 53, 54. While more clinical trials should be conducted in order to further confirm the protective effects of metformin in heart failure, studies discussed above suggest that metformin has therapeutic potential for this disease. In addition, results from several animal studies revealed that metformin benefited patients with pulmonary hypertension associated with heart failure with preserved ejection fraction to some extent 55, 56.

Aging-related cardiovascular diseases are often caused by a combination of multiple factors, which include telomere shortening and mitochondrial oxidative stress in vascular endothelial cells as well as cardiomyocyte dysfunction and other factors. Preclinical studies show that the protective effect of metformin on cardiovascular diseases is based on its multiple effects on vascular endothelial cells, smooth muscle cells, lipids and chronic systemic inflammation 57, 58. For example, metformin was reported to improve the function of vascular smooth muscle cells by inhibiting inflammation, contraction, proliferation and calcification 58. Of note, the study to explore the direct effect of metformin on the senescence of vascular smooth muscle cells is still lacking.

### 3.5 Neurodegenerative diseases

Neurodegenerative diseases mainly include Alzheimer's disease (AD), Huntington's disease (HD), and Parkinson's disease (PD). The common pathological feature of neurodegenerative diseases is the presence of a large number of misfolded and aggregated proteins in neurons, such as mutated α-co-nucleoprotein, β-amyloid, tau protein, and Huntington's protein. These proteins have toxic effects on neurons, thus affecting neuronal connectivity and plasticity, and may even trigger the activation of cell death signaling pathways, leading to massive neuronal death. Of importance, aging is a main risk factor of neurodegenerative diseases 59.

The efficacy of metformin in the treatment of AD seems to be controversial. Most of the current evidence suggests a beneficial effect of metformin on the prevention of AD, including improved cognitive performance and decreased risk of AD. A large meta-analysis revealed that patients with T2D using metformin displayed a reduced risk for developing AD 60. Similarly, results from a prospective observational study support the notion that metformin slows cognitive decline in old patients with diabetes 61. However, results from a case-control study by Imfeld et al. revealed that long-term use of metformin increased the risk of AD 62. In addition, Moore et al. reported that diabetic patients developed worse cognitive performance after treatment with metformin 63. The efficacy of metformin in the treatment of PD is also controversial. Results from a retrospective study by Wahlqvist et al. revealed that metformin reduced the risk of PD in T2D patients in a Taiwanese population 64. In contrast, results from another cohort study showed that metformin treatment was associated with an increased risk of PD in T2D patients 65. The controversy may be due to differences in sample sizes, statistical methods and applications of drug combination from these studies. Metformin treatment for HD is still at the preclinical stage, and results from some animal models showed that metformin was beneficial to some extent in the treatment of HD 66.

### 3.6 Cancers

Aging plays an important role in causing cancers. Current studies suggest that metformin can be used alone or in combination with other drugs in cancer treatment, including pancreatic, breast, colon and other cancers 67.

Results from several retrospective studies and meta-analyses suggested that metformin treatment increased survival in patients with pancreatic ductal adenocarcinoma (PDAC) 68, 69. However, result from one study showed that the addition of conventional antidiabetic doses of metformin did not improve prognosis in patients with advanced PDAC treated with gemcitabine and erlotinib 70. The different selection of patient recruitment and statistical analytical method may contribute to the controversial results. The protective effect of metformin against breast tumors and colon cancer is clear. A study by Bodmer et al. showed a reduced risk for breast cancer in women with T2D by long-term metformin usage 71. Further studies showed that the protective effect of metformin against breast cancer was related to the types of the breast cancers and the hormone levels in patients. Metformin reduced the serum level of estradiol in patients, a possible mechanism by which metformin resists breast cancer development 72. Meanwhile, another study reported that T2D patients on long-term metformin use showed reduced risk of estrogen receptor-positive breast cancer 73. Several clinical studies reported that metformin application in T2D patients not only reduced the risk of colorectal cancer, but also improved the survival rate of colorectal cancer patients 74, 75. In addition, metformin was shown to reshape the methylation profile of colon cancer cells, which may contribute to its anti-colon-cancer effects 76. In addition to the tumors discussed above, the anti-cancer effects of metformin have been demonstrated in other tumors. Wang et al. showed in a population-based cohort study that the use of metformin reduced the risk of esophageal cancer and that the effect was significant in new users and the users aged 60-69 years 77. A regression study showed a negative association between metformin use and the incidence of lung cancer and mortality in lung cancer patients 78. Some preclinical studies also suggested anti-cancer effect of metformin in other cancers, such as bladder cancer 79, however, more clinical studies are necessary to verify it.

Metformin exerts its anti-cancer effect mainly by impeding tumor cell proliferation, blocking the tumor cell cycle progression, preventing the genomic instability, inducing apoptosis and impacting cellular energy metabolism (Table 2). Of note, there still are limitations in the current studies on the anti-tumor mechanisms of metformin, which should be investigated by further preclinical studies.

### 3.7 Obesity and other metabolic abnormalities

Obesity is a public health problem worldwide. As a multifactorial chronic disease, obesity is often accompanied by metabolic abnormalities, such as T2D, fatty liver, and cardiovascular disease. The prevalence of obesity has markedly risen among adults over 65 years old 80. A growing body of evidence suggests that metformin is a potential treatment for obesity and its related metabolic dysfunction. In humans, metformin displayed a significant effect on weight loss in patients who gained weight as a result of antipsychotic treatment 81.

Above result is also supported by increasing preclinical evidence. Kim and coworkers reported that metformin increased the expression of fibroblast growth factor 21 in high-fat-diet (HFD) fed mice, thereby preventing obesity and inflammation caused by HFD 82. In addition, Geerling et al. reported that metformin prevented obesity in mice by promoting the metabolic activity of mitochondria-rich brown adipose tissue 83. In addition, metformin was reported to regulate the intestinal microbiota and prevented obesity caused by HFD in rats 84. Recent studies reveal that aging and obesity are closely related to the loss of stemness properties in adipose stem cells. Chinnapaka et al showed that metformin improved the stemness of human adipose-derived stem cells by downregulating the mTOR and ERK signaling 85. Age-related adipose tissue dysfunction caused by stress-induced senescence of adipose stromal cells was ameliorated by metformin treatment 86.

As an effective anti-glycemic agent, metformin plays an important role in regulation of glucose metabolism. Metformin inhibited gluconeogenesis in liver. Results from a number of studies reveal that metformin exerts its hypoglycemic effect through activation of the AMPK 87, 88. Zhou et al. showed that metformin activated AMPK in hepatocytes 89. HFD-fed mice with liver selective deletion of AMPKα1/2 subunits showed an increase in their blood glucose level when they were treated with metformin 90. Metformin failed to improve the hyperglycemia in mice lacking serine-threonine liver kinase B1 (LKB1) in liver 91. These studies support a mechanism through which metformin exerts its hypoglycemic effect through activation of the LKB1-AMPK pathway. However, the importance of AMPK in mediation of the effect of metformin on hepatic glucose production (HGP) is currently being challenged. Foretz et al. reported that the blood glucose levels in mice lacking AMPK in the liver were comparable to those in wild-type mice, and the hypoglycemic effect of metformin was intact in these mice 92. In addition, a previous study reported that metformin non-competitively inhibited the redox shuttle enzyme mitochondrial glycerophosphate dehydrogenase, which altered in the redox state in liver cells and reduced the conversion of lactic acid and glycerol to glucose and thereby impairs liver gluconeogenesis 93, 94. These findings indicate that mechanisms underlying the metformin's HGP and hypoglycemic effects in diabetes are complicated, which involve multiple pathways.

In addition to its important role in glucose metabolism in liver, metformin also plays roles in other organs. For example, in the intestine, metformin was shown to relocate GLUT2 by mediating AMPK activation and promote glucose uptake in the intestinal mucosa 95. Several studies showed that OCT3 was expressed in fat cells and muscle cells and transported metformin into these cells 96. Lin et al. reported that metformin improved fatty liver, reversed steatosis and abnormal transaminase 97. Kita et al. reported that metformin prevented and reversed the steatosis of nonalcoholic steatohepatitis in experimental non-diabetic models without affecting peripheral insulin resistance 98. In addition, metformin inhibited adipogenesis in adipocytes, which was abolished in acetyl CoA Carboxylase 1/2 mutant mice 99. Metformin inhibited fatty acid desaturase and thereby reduced the levels of acyl-alkyl phosphatidylcholines and LDL-C 100. Collectively, these studies suggest that metformin play an important role in regulation of liver lipid metabolism.

Metformin also regulates amino acid and protein metabolism. In a 2016 study, the authors reported results from individuals treated with metformin for 18 months and analyzed every 6 months. They found that metformin reduced the levels of phenylalanine and tyrosine in the blood and increased the levels of branched chain amino acids, histidine and alanine levels 101. Subsequent studies from the same group revealed that metformin increased the blood levels of leucine, isoleucine and tyrosine in patients with diabetes and insulin resistance 102, 103. While these studies indicate that metformin regulates metabolisms of amino acids and proteins, its underlying mechanisms remain to be defined.

### 3.8 Other diseases

Metformin has been used to treat other diseases, including polycystic ovarian syndrome (PCOS) and COVID-19. In pregnant women with PCOS, metformin treatment from the late first trimester until delivery reduced the risk of late miscarriage and preterm birth, but failed to prevent gestational diabetes 104. Virus-induced cellular senescence (VIS), a common response of host cells to viral stress, is an important manifestation of SARS-CoV-2 infection and has been demonstrated in several dimensions. Several senolytic drugs, such as bafilomycin and quercetin, were shown to counteract cellular senescence caused by SARS-CoV-2 105. Metformin prevented cellular senescence and it suggested that patients with COVID-19 might be benefited from metformin 105. Results from several studies showed that patients with diabetes needed a longer recovery time after being infected with COVID-19 and experienced longer-lasting adverse reactions sequelae in multi organs. Moreover, infection with SARS-CoV-2 was reported to promote onset of new diabetes or aggravation of the original metabolic disorder 106. Several retrospective studies demonstrated that metformin reduced the mortality of patients no matter whether they were diabetic or not 107, 108. However, results from a retrospective study revealed that in individuals with COVID-19 and preexisting T2D, the use of metformin was associated with an increased incidence of acidosis, suggesting that the basic underlying disease of patients with COVID-19 should be considered before the usage of metformin 109. While mechanism(s) underlying metformin's effects on VIS remain poorly defined, it is believed that metformin use can decrease risk of death for patients with COVID-19.

Above evidence from clinical and preclinical studies suggests the beneficial effects of metformin in attenuating aging and treating aging-related diseases. Several clinical trials have been designed and performed to further evaluate the effectiveness of metformin on molecular and clinical outcomes of individual hallmarks of aging (Table 3), knowledge from these trials may facilitate the application of metformin in the treatment of aging and aging-related diseases.

## 4. Molecular mechanisms and signal transduction pathways

**Figure 3:**
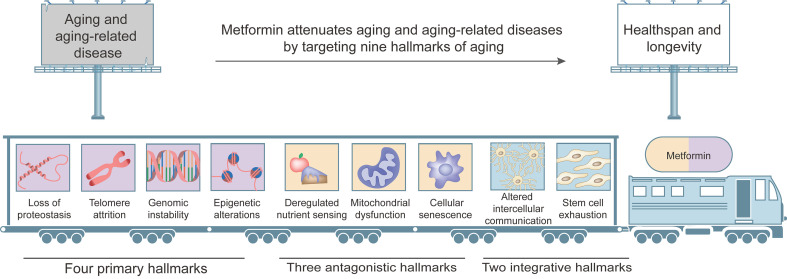
** Targets of metformin among the hallmarks of aging.** Metformin attenuates aging and aging-related diseases by targeting nine hallmarks of aging, including (1) four primary hallmarks (loss of proteostasis, telomere attrition, genomic instability and epigenetic alterations); (2) three antagonistic hallmarks (deregulated nutrient sensing, mitochondrial dysfunction, and cellular senescence); (3) two integrative hallmarks (altered intercellular communication and stem cell exhaustion).

Cumulative evidence from a number of experiments and clinical trials demonstrates that metformin delays aging and has significant protective effects against aging-related diseases mainly by affecting hallmarks of aging, including dysregulated nutrient sensing, loss of proteostasis, mitochondrial dysfunction, altered intercellular communication, telomere attrition, genomic instability, epigenetic alterations, stem cell exhaustion and cellular senescence (Figure 3).

### 4.1 Dysregulated nutrient sensing

**Figure 4:**
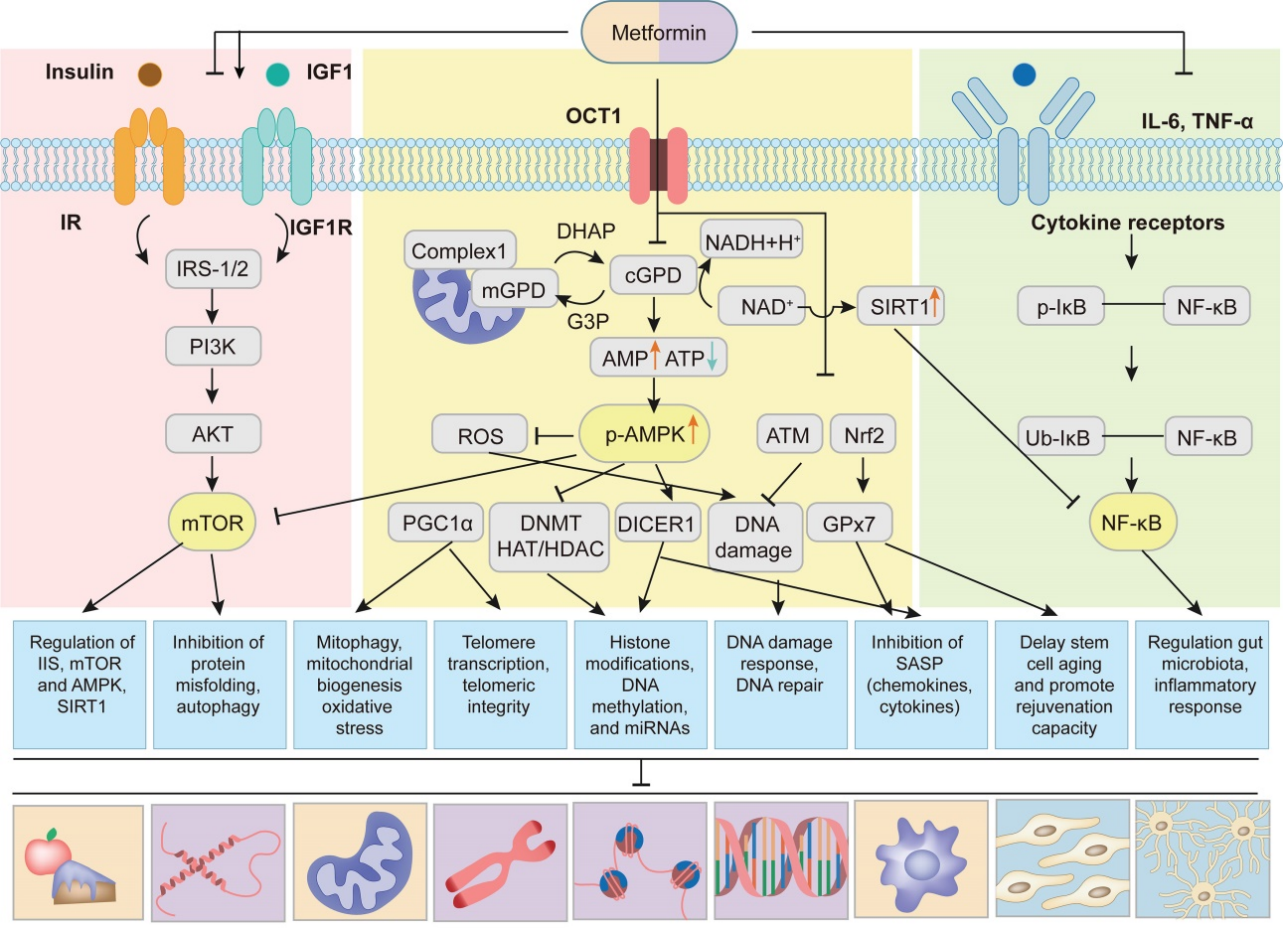
** Multiple pathways of metformin targeting aging and aging-related diseases.** Metformin alleviates aging and aging-related diseases by targeting aging hallmarks through following signaling pathways: (1) Metformin is transported into cells through organic transporter 1 (OCT1). Then metformin inhibits mitochondrial respiratory-chain complex 1 and thereby oxidative phosphorylation, resulting in increased AMP/ATP and NAD^+^/NADH ratios, causing activation of AMPK and up-regulation of SIRT1. AMPK-dependent mechanisms lead to the inhibition of mTOR, reactive oxygen species (ROS), DNA Methylation (DNMT) and histone acetyltransferase (HAT)/ histone deacetylase (HDAC), and the increase of PGC1α and DICER1. Metformin also up-regulates ataxic telangiectasis mutation (ATM) protein kinase and nuclear factor erythroid 2-related factor 2 (Nrf2), contributing to the inhibition of DNA damage and increase of glutathione peroxidase 7 (GPx7). (2) Metformin regulates the insulin and IGF-1 signaling and thereby the phosphorylation of insulin receptor substrate-1/2 (IRS-1/2) and PI3K/AKT/mTOR signaling. Activation of AMPK also inhibits mTOR signaling. (3) Metformin inhibits NF-κB signaling induced by pro-inflammatory cytokines. Up-regulation of SIRT1 further inhibits NF-κB signaling. Adapted from Kulkarni et al^7^.

Metabolic homeostasis is an essential part of the cellular and organismal homeostasis. Studies by Bettedi et al. demonstrated that multiple signaling pathways that sense energy status and nutrients availability of cells communicated with growth factor and growth hormone signaling pathways to orchestrate metabolic homeostasis in humans 110. Smith et al showed that during aging the nutrient sensing pathways were dysregulated, which resulted in a gradual deterioration in the regulation of metabolic homeostasis 111. Studies from several groups revealed that metformin exerted its anti-aging effects by improving the dysregulated nutrient sensing, mainly through regulation of four most important nutrient-sensing signaling pathways, including down-regulation of the insulin/insulin-like growth factor 1 (IGF-1) and mTORC1 signaling pathways and up-regulation of AMPK and SIRT1 signaling pathways 5, 7 (Figure 4). Metformin decreased the levels of insulin and IGF-1, and thereby inhibited phosphorylation of insulin receptor substrate-1/2 (IRS-1/2) and PI3K/AKT/mTOR signaling, which plays a key role in controlling aging and cancer 7, 112. It is worth noting that metformin could also up-regulate IRS/ PI3K/AKT signaling to improve glucose metabolism and cognitive impairment 113, 114. The discrepancies of the metformin actions may result from the complex regulation mechanisms of IRS-1/2 signaling pathway, including positive and negative feedback loops 114. The mTOR kinase is composed of mTORC1 and mTORC2. Studies using genetic models demonstrated that it was the down-regulation of mTORC1 rather than that of mTORC2 that extended longevity 115. Kalender et al. showed that metformin directly inhibited mTORC1 in Ras-related GTP-binding protein (Rag) GTPase-dependent manner 116. In addition, van Nostrand and coworkers reported that metformin inhibited mTORC1 by AMPK activation-mediated phosphorylation of Raptor and tuberous sclerosis complex 2 (TSC2) 117. Sirtuin family has the function of ADP ribosyltransferases and NAD-dependent protein deacetylases and plays a vital role in energy metabolism. The Sirtuin isoforms (SIRT1-SIRT7) have been extensively studied and considered as potential and attractive anti-aging factors. Recent studies revealed that in low NAD^+^ concentrations, metformin directly activated SIRT1 and enhanced anabolic signaling, thus favoring healthy aging 118. Interestingly, more recent study 119 found that metformin didn't influence the abundance of proteins linked with these nutrient sensing pathways and aging. This might be due to the regulation of metformin on the phosphorylation of nutrient sensing pathways instead of abundance, which needs to be confirmed in the further study.

### 4.2 Loss of proteostasis

Protein homeostasis or proteostasis is important for cell function and viability. Under normal physiological conditions, cells take advantage of a series of processes to regulate the synthesis, folding, conformational stability and degradation of their proteomes to maintain proteostasis. Proteostasis involves mechanisms for the stabilization of correctly folded proteins by chaperone-mediated protein folding and stability system, and mechanisms for the degradation of proteins by the autophagy-lysosomal system and the ubiquitin-proteasome system. However, aging and aging-related diseases are usually accompanied with loss of proteostasis. Metformin attenuates aging and aging-related diseases by maintaining proteostasis through inhibition of protein misfolding and enhancement of autophagy 120. In a D-galactose-induced aging rat model, metformin was shown to maintain protein homeostasis by regulating unfolded protein response (UPR)-related chaperone proteins, including heat shock protein (HSP) 60, HSP90, glucose-regulated protein 78 (GRP78) and C/EBP homologous protein, and alleviate aging‑related hearing loss and neurodegeneration 121. In an Alzheimer disease mouse model, Xu and coworkers showed that metformin promoted the activation of chaperone-mediated autophagy and partly reversed the pathologies of this aging-related disease 122. Further, recent studies showed that metformin could ameliorate inflammation by stimulating expression of Krüppel-like factor 2 (KLF2) 123, 124 and autophagy 125 in different cell types (such as T cells, endothelial cells and macrophages) 57, which highlight the important role of metformin-induced autophagy in the alleviation of aging-associated inflammation.

### 4.3 Mitochondrial dysfunction

Mechanisms of mitochondrial dysfunction and aging remain poorly understood. Mitochondria are important organelles that provide energy and maintain homeostasis in cells. Abnormalities in mitochondrial function caused by various pathological factors affect cell metabolism and impair body function and health. Results from a study by Konopka et al. showed that metformin improved the physiological functions of the elderly by eliminating exercise-mediated increases in skeletal muscle mitochondrial respiration 126. Metformin up-regulated mitochondrial biogenesis by SIRT3 through AMPK-mediated H3K79 methylation, and delayed aging 127. Increased levels of reactive oxygen species (ROS) and the imbalance of energy metabolism caused by the decline of mitochondrial function were shown to be related to the occurrence of aging and a variety of aging-related diseases 128, and may be targeted for the treatment of aging-related diseases 129.

Recent studies reported that metformin reduced ROS production by mitochondrial complex I (NADH: ubiquinone oxidoreductase) and improved oxidative stress-mediated dysfunction or cell apoptosis 130. PGC-1α is a co-transcriptional regulator that promotes mitochondrial biogenesis. A large number of studies have shown that metformin can improve mitochondrial biogenesis by increasing the expression of PGC-1α in a tissue-specific manner. Suwa et al. showed that metformin enhanced PGC-1α expression and mitochondrial biogenesis by promoting AMPK phosphorylation and improving insulin resistance in skeletal muscle 131. Andrzejewski et al. showed that the elevated level of PGC-1α in breast cancer cells helped to cope with the interference of metformin on mitochondrial energy metabolism, and played roles in drug resistance and metastasis 132. Metformin selectively affected PGC-1α mediated gene regulation in the liver and prevented gluconeogenesis activation, but did not affect its regulation of mitochondrial genes 133. In addition to mitochondrial dysfunction, recent studies suggested that the therapeutic effect of metformin on aging-related diseases might be through regulation of mitochondrial dynamics, including mitochondrial fission and fusion 134, 135. Whether mitochondrial fission proteins (such as dynamin-related protein 1 and mitochondrial fission protein 1) and mitochondrial fusion proteins (such as optic atrophy 1, mitofusin-1 and mitofusin-2) play important roles in the anti-aging effect of metformin remains unclear and requires further investigation.

### 4.4 Altered intercellular communication

Aging not only impacts the cell autonomous system, but also affects other cellular functions through intercellular communication, such as neuronal, endocrine and neuroendocrine, which tends to lead to chronic, low-grade and sterile inflammation in the body. Inflammation promotes epigenetic changes, loss of protein stability and stem cell dysfunction, and is a key factor leading to aging-related diseases. In addition to regulating metabolic indicators, metformin also reduces the level of low-grade inflammation in the blood 136. Clinical follow-up showed that metformin alone significantly reduced circulating pro-inflammatory cytokine levels and mortality in elderly diabetic patients 137. Metformin inhibited the expression of pro-inflammatory cytokines, such as TNF-α and IL-6, by regulating the NF-κB pathway, and reduced the susceptibility to aging-related diseases 138. Gut microbiota is closely related to metabolism and immune homeostasis 139. Aging-related gut microbiota imbalance was shown to lead to the release of inflammatory factors and increase levels of inflammation in the body through intercellular communication 140, 141. Metformin regulated metabolism-related diseases by regulating the composition of the gut microbiota 142-144, such as improving metabolism, reducing body weight and indirectly reducing the level of systemic inflammation. Metformin may be beneficial in improving aging and aging-related diseases by regulating gut microbiota and inhibiting inflammation.

### 4.5 Telomere attrition

Telomeres located at the ends of chromosomes are essential for protecting the integrity of chromosomes and controlling organismal aging. It is known that biological aging and aging-related diseases are associated with telomere attrition and shortening. In addition, telomere damage or dysfunction caused by various pathological factors can accelerate aging, increase the incidence of aging-related diseases, and even shorten life span 145. Gao et al. showed aging-related telomere attrition in adipocyte progenitors predisposed to metabolic diseases through human biopsies and gene knockout mice 146. In leucocytes, relative telomere length was shown to be inversely associated with glycemic level in T2D patients diagnosed in less than 5 years and was associated with a higher incidence of diabetic complications 147. Activation of telomerase by pharmacology or transgenic technology reversed the premature aging phenotype of telomerase deficient mice or delay physiological aging 148, 149. AMPK was shown to regulate telomere transcription through telomere repeat RNA 150. Metformin reduced telomere attrition of leukocytes in patients with mild aging-related diabetes and had obvious anti-aging effect 151. Garcia-Martin showed that metformin therapy prevented telomere shortening associated with leukocyte and placental telomere shortening in patients with gestational diabetes mellitus, thereby avoiding adverse fetal outcomes 152.

### 4.6 Genomic instability

Genomic instability is mainly due to DNA damage and mutation accumulation caused by multiple endogenous (mitochondrial metabolism) and exogenous (environmental) factors, and can be offset by the large DNA repair network. Genomic instability is a key marker of aging, and artificially induced genomic instability accelerates cell aging and increases susceptibility to aging-related diseases 153.

It is believed that metformin protects genomic stability by in part inhibiting oxidative stress and DNA damage and by regulating ataxic telangiectasis mutation (ATM) protein kinase. Metformin inhibited DNA damage in Drosophila by down-regulating aging-related and oxidative stress-induced AKT activity 154. In T2D patients, the antioxidant capacity of metformin protected cells from diabetic oxidative stress and regulated DNA BER system, which was related to the significant up-regulation of X-ray repair cross complementing 1 and p53 levels 155. In addition, metformin inhibited hepatic gluconeogenesis by regulating ataxic telangiectasis mutation and activating multiple AMPK-dependent and AMPK-independent differentially expressed genes and regulatory elements 156. Vazquez-Martin and coworkers showed that metformin recruited DNA repair complexes to repair broken DNA double strands by activating ATM and Checkpoint Kinases-2 157. Metformin improved homologous recombination defect tumor-dependent DNA repair by regulating metabolic levels 158. Although the potential mechanism of metformin's protection of genome stability is not fully elucidated, its antioxidant and DNA damage prevention effects are beneficial to the prevention and treatment of aging-related disease damage.

### 4.7 Epigenetic alterations

Alterations in epigenetic modifications, such as histone modification and DNA methylation and non-coding RNAs, can promote inflammation and metabolic aging phenotypes, leading to a variety of aging-related diseases. Restoring the normal state of epigenetic characteristics may be a potential therapeutic strategy for the treatment of aging and aging-related diseases.

Increasing evidence suggests that metformin can regulate transcription and post-transcriptional activity through distinct epigenetic modification mechanisms 159. It was reported that abnormal methylation of the whole genome in some T2D patients caused metformin intolerance 160. Metformin promoted global DNA methylation through the mitochondrial one-carbon metabolic pathway and H19/S-adenosine homocysteine hydrolase axis, and regulated expression of epigenetic genes to promote health 161, 162. *In vitro* studies showed that metformin reduced histone H3 lysine 27 trimethylation (H3K27me3) and inhibited the activity of ovarian cancer cells, reflecting its anti-tumor effect by regulating epigenetic modification 163. *In vivo*, metformin reversed the physiological function of histone H3K36me2 in 170 histone markers that is known to play a role in pathogenesis of diabetes and obesity in prediabetic HFD-induced obesity mouse models 164.

In addition to its effects on DNA and histone methylation, metformin affects cell function and intervenes in related diseases by regulating non-coding RNAs expression. Metformin was reported to reduce cell senescence and delay aging-related phenotypes by regulating miRNA expression in several *in vitro* and *in vivo* aging models 165, 166. Results from clinical study showed that metformin might regulate aging-related metabolic and non-metabolic pathways in skeletal muscle and subcutaneous adipose tissue of older adults by controlling miRNA-29b expression 19. In addition, metformin was shown to prevent vascular smooth muscle cell dysfunction by regulating long non-coding RNAs (lncRNA) or circular RNAs (circRNAs) 167, 168. However, the precise mechanisms how these non-coding RNAs participate in the metformin actions still need to be sufficiently understood.

### 4.8 Stem cell exhaustion

Stem cells are unspecialized cells, which exist in all stages of life, such as embryo, fetus and adult. These cells can give rise to various differentiated cells that contribute to the properties of different tissues and organs 169. At the early postnatal and adult stages of human life, tissue-specific stem cells are present in differentiated organs and play key roles in tissue repair and regeneration following injury to the organ. With aging, stem cells are gradually exhausted and their biological behaviors, such as viability, proliferation, differentiation and migration, are inhibited, which leads to an impaired tissue regenerative capacity and results in tissue and organ dysfunction as well as aging-related diseases. Increasing evidence suggests that metformin delays stem cell aging and activates its rejuvenation capacity by increasing antioxidant effect, activating AMPK signaling, and inhibiting mTORC1 to initiate autophagy. Low-dose metformin administration was shown to upregulate the expression level of endoplasmic reticulum-localized glutathione peroxidase 7 (GPx7) via nuclear factor erythroid 2-related factor 2 (Nrf2), which delayed premature cellular senescence and extended the lifespan of human mesenchymal stem cells 170. Results from Neumann and colleagues showed that metformin similarly activated AMPK signaling and rejuvenated aging in oligodendrocyte progenitor cells 171. Moreover, in Drosophila midgut intestinal stem cells, Na et al. 172 demonstrated that metformin alleviated aging-related phenotypes by regulating autophagy related gene 6 and AKT/mTOR signaling pathway.

### 4.9 Cellular senescence

Cellular senescence is usually defined as an irreversible arrest of the cell cycle, which can be induced by various internal and external stressors, including irradiation, mitochondrial dysfunction, oxidative and genotoxic stress, oncogenic activation and chemotherapeutic agents. Besides cell cycle arrest, senescent cells often show other phenotypic alterations, such as chromatin rearrangement, metabolic reprogramming and autophagy modulation. Senescent cells can produce a complex mixture of chemokines, cytokines, growth factors and extracellular matrix proteases, which are collectively called senescence-associated secretory phenotype (SASP). Cellular senescence is widely considered to be a main driver of aging and aging-related diseases, with the accumulation of senescent cells and the inflammatory microenvironment created by SASP via paracrine and autocrine. Metformin was shown to inhibit SASP and cellular senescence in multiple age-related dysfunctions 7, 173. In lens epithelial cells and nucleus pulposus cells, metformin reduced SASP and senescence by activation of AMPK signaling and restoration of autophagic flux 39, 174. In renal tubular epithelial cells, metformin inhibited senescence and alleviated diabetic nephropathy through the muscleblind-like 1 /miR-130a-3p/STAT3 signaling pathway 175. In addition, metformin repressed endothelial senescence by activating the activity of Sirt1 176, and inhibited macrophage senescence and SASP by down-regulating NLRC4 phosphorylation 177. Śmieszek A et al. 178 found that metformin suppressed SASP and senescence in *ex vivo* cultures of murine olfactory ensheathing cell via down-regulation of NF-κB signaling. Interestingly, several studies reported that metformin increased SASP and senescence in cancer cells 179, 180. These contradictory results suggest that mechanisms whereby metformin regulates SASP and cellular senescence are complicated, which need more detailed investigation.

## 5. Conclusion and perspectives

Metformin has significant effects in anti-aging and in attenuating aging-related diseases and displays promising perspective in aging-related clinical applications. However, there are several important issues that need to be addressed. First, more multicenter, large-scale, double-blind, randomized, placebo-controlled trials are required to further elucidate the effects of metformin on aging and major aging-related diseases. Second, the concept of precision or personalized medicine should be considered in the anti-aging treatment of metformin. Even though metformin has become the first-line medicine to treat T2D, there are non-responders and responders, and side effects also vary considerably. It is important to take into account the patients' genetic background, the dose of medicine and the approach of administration in particular, and then develop personalized treatment strategy. Third, there is some limitations of the administration of metformin in clinic because of its short half-life and relatively low bioavailability. The design of novel drug delivery systems to improve drug bioavailability, enhance drug stability and reduce side effects of metformin are largely appreciated in clinical applications. Finally, distinct molecular mechanisms through which metformin attenuates aging and different aging-related diseases are still poorly understood and remain to be investigated in great detail. Better understanding of these mechanisms will greatly facilitate the development of novel and efficient strategies for the prevention and treatment of aging and aging-related diseases by metformin.

## Figures and Tables

**Table 1:** Preclinical and clinical results of metformin in targeting aging and aging-related diseases.

Aging and aging-related diseases		Preclinical and clinical results
Aging and lifespan		Regulate the metabolism of HEK293T through lysosomal pathway 20; Extend the lifespan of C. elegans and mice 20, 21; Reduce all-cause mortality 17-19.
Diabetes mellitus		Change the redox state of liver cells and reduce hepatic gluconeogenesis 93; Reduce blood glucose in mice 92; Improve blood glucose control and lipid concentration in patients with non-insulin-dependent diabetes mellitus (NIDDM) 24, 181.
Degenerative musculoskeletal diseases	Osteoporosis	Enhance the differentiation and mineralization of osteoblast and inhibits osteoclast differentiation, prevent bone loss in ovariectomized rats 182, 183; Metformin is associated with a lower risk of fracture 28, 29.
	Osteoarthritis	Inhibit the expression of inflammatory factors, matrix metalloproteinases and hypertrophy markers in chondrocytes through bone marrow stromal stem cells in co-culture model 33, 36; Delay the progression of osteoarthritis and reduce pain in primate and mice with osteoarthritis 35, 36; Protect joint by anti-inflammatory, regulating skeleton and reducing weight 31, 33, 34.
	Intervertebral disc degeneration	Protect nucleus pulposus cells against apoptosis and senescence or exert an anti-inflammatory effect; Reduce local mechanical hyperalgesia in the nucleus pulposus and annulus fibrosus 39, 40.
	Other bone disorders	Increase glycolytic activity and decrease the expression of inflammatory factors primary synovial fibroblast 43; Decrease the inflammatory response, oxidative stress and bone loss in ligature-induced periodontitis in rats 44; Prevents against oxidative stress-induced senescence in human periodontal ligament cells 45; Inhibit the ossification and inflammation of ankylosing spondylitis fibroblasts 46; Inhibit the proliferation and metastasis of osteosarcoma cells 47.
Cardiovascular disease	Coronary heart disease	Protect hyperglycemia-induced endothelial impairment 184; Reduce coronary endothelial dysfunction and early progression of coronary plaque 49, 51.
	Heart failure	Reduce myocardial oxygen consumption and left ventricular mass index, left ventricular mass, office systolic blood pressure and oxidative stress in patients 53-56.
	Heart attack combined with pulmonary hypertension	Benefit rats in a rodent model of metabolic syndrome and pulmonary hypertension associated with heart failure with preserved ejection fraction 55, 56.
Neurodegenerative diseases	Alzheimer's disease	Prevent patients from Alzheimer's disease^60^, ^61^ or increase the risk of it 62, 63.
	Huntington's disease	Rescue the motor and neuropsychiatric phenotypes of Huntington's disease in zQ175 knock-in mouse 185.
	Parkinson's disease	Reduce the risk of Parkinson's disease 64, or increase the risk of PD in T2D patients^65^.
Obesity and other metabolic abnormalities	Obesity	Inhibit adipogenesis in adipocytes 46; Prevent obesity in mice 82, 83 and rats 84; Promote weight loss in patients who gain weight as a result of antipsychotic treatment 81.
	Fatty liver disease	Improve fatty liver disease, reversing steatosis and aminotransferase abnormalities 97.
Other diseases	Polycystic ovary syndrome	Induce ovulation in women with the polycystic ovary syndrome 186.
	Chronic kidney disease	Metformin could have benefits on kidney disease progression and may lower the risk of death 187.
	COVID-19	Reduce the Mortality of patients with COVID-19 107, 108.
	Hidradenitis suppurativa	Metformin is an effective treatment option for hidradenitis suppurativa 108.

**Table 2:** Effect of metformin in targeting cancers.

Types of cancer	Research object	Effects	References
Bladder cancer	Human cell lines (UMUC3 and J82 cells)	Block bladder cancer growth and survival through SREBP-1c/FASN axis by targeting the expression of clusterin.	79
Lung cancer	Human (Clinical research)	Associated with low risk of lung cancer; Co-treatment with EGFR-TKIs therapy could improve progression-free survival in patients with advanced lung adenocarcinoma.	78, 188
Colorectal cancer	Human (Meta-analysis)	Reduce the risk of colorectal cancer and improve the survival rate of colorectal cancer patients.	75
Leukemia	Human cell lines (MMCLs)	Co-treatment with bortezomib could enhance the anti-myeloma effect of it leading to delaying the growth of myeloma xenotransplants.	189
Breast cancer	Human (Clinical research)	Metformin could reduce the risk of breast cancer based on patient's hormone levels.	71, 72
Esophageal cancer	Human (Clinical research: cohort study)	Metformin use decreases the risk of developing esophageal cancer, especially in new metformin users and participants aged 60-69 years.	77
Pancreatic ductal adenocarcinoma	Human (Clinical research)	The effect of metformin to treat pancreatic ductal adenocarcinoma is mixed.	68-70
Endometrial cancer	Human (Clinical research: Phase 2 Randomized Clinical Trial)	Co-treatment with everolimus, letrozole could result in a higher rate of clinical benefit for women with advanced or recurrent endometrial cancer.	190
Melanoma	Human cell lines (A2058 and A375)	Inhibit melanoma cancer cell motility and growth through inducing cell cycle arrest and promoting cell apoptosis.	191
Thyroid Cancer	Human cell lines (FTC133 and BCPAP)	Inhibit growth of thyroid cancer cells by downregulating the expression of mGPDH and inhibiting OXPHOS *in vitro* and *in vivo*.	192
Osteosarcoma	Human and mice cell lines (MG63 and K7M2)	Suppress the self-renew of osteosarcoma stem cells through ROS-mediated apoptosis and autophagy.	193
Primary bone cancer	Human (Clinical research: retrospective cohort study)	Reduce the risk of primary bone cancer in men with T2D aged more than 60 years.	194
Hepatocellular carcinoma	Human cell lines (HepG2 and Huh7 and 293FT)	Suppress the growth and increase cell death of hepatocellular carcinoma cells by elevating oxidative phosphorylation.	195
Gastric Cancer	Human (Clinical research)	Decrease the cancer-specific mortality rates, recurrence and all-cause mortality of gastric cancer patients with diabetes who underwent gastrectomy.	196
Ovarian Cancer	Human cell lines (SKOV3, OVCAR3 and HO8910)	Induce cancer cells apoptosis through triggering endoplasmic reticulum stress.	197
Kidney cancer	Human (Clinical research: cohort study)	Reduce the risk of kidney cancer in patients with type 2 diabetes.	198
Prostate cancer	Human (Clinical research: cohort study)	Reduce the risk of prostate cancer among men with type 2 diabetes.	199

**Table 3:** Clinical trials using metformin for targeting biological aging.

NCT number	Title	Conditions	Characteristics of biological aging (Outcome Measures)
NCT03309007	A Double-Blind, Placebo-Controlled Trial of Anti-Aging, Pro-Autophagy Effects of Metformin in Adults with Prediabetes	PreDiabetes, Aging	Change in leucocyte LC3 score
NCT04994561	VIAging Deceleration Trial Using Metformin, Dasatinib, Rapamycin and Nutritional Supplements	Aging	Senescent cell-cycle arrest physiological parameter value measured by MMP-9 laboratory test; Glucose control (insulin resistance) physiological parameter as measured by HOMA-IR (mg/dL) calculation value; and etc.
NCT02432287	Metformin in Longevity Study (MILES)	Aging	Increase in number of expressed genes in muscle and adipose tissue using RNA sequencing; Mixed meal tolerance. Assessment of insulin sensitivity and insulin secretion
NCT02308228	Metformin to Augment Strength Training Effective Response in Seniors (MASTERS)	Aging	Percent change in type 2 myofiber cross sectional area; Percent change in normal density muscle size by computed tomography
NCT04264897	Antecedent Metabolic Health and Metformin Aging Study	Aging, Insulin Sensitivity, Chronic Disease, Mitochondria, Insulin Resistance	Mean change in insulin sensitivity measure, mitochondrial function of the electron transport system measured by complex I activity, daily average glucose measure, and blood-based biomarker measures of aging
NCT03451006	Effect of Metformin on Frailty in 12 Subjects	Aging, Inflammation, Frailty	Change in frailty, interleukin 6 (pg/mL), matrix metalloproteinase (ng/mL), plasminogen activator inhibitor, monocyte chemotactic protein-1, activin, and etc.
NCT03713801	Impact of Metformin on Immunity	Aging, Vaccine Response Impaired	Change in antibody responses to PCV13; Measure of immunophenotypes
NCT03072485	Phase 1 Study of the Effects of Combining Topical FDA approved Drugs on Age-related Pathways on the Skin of Healthy Volunteers	Aging	Profile of gene transcript changes; Wrinkle score
NCT03996538	Vaccination Efficacy with Metformin in Older Adults	Aging, Age-Related Immunodeficiency, Vaccine Response Impaired	Change in cell-mediated flu vaccine responses, Cell-mediated flu vaccine responses, influenza antibody titers, T cell metabolic phenotype, T cell oxygen consumption rate, frailty phenotype
NCT02745886	Metformin Induces a Dietary Restriction-like State in Human	Overweight Subjects, Metformin, Aging	The differences of gene expression profile among 3 groups, insulin sensitivity in 3 groups
NCT04221750	Diet and Exercise Plus Metformin to Treat Frailty in Obese Seniors	Frailty, Sarcopenic Obesity, Aging	Change in the modified physical performance test, muscle strength, dynamic balance, static balance, gait speed, peak aerobic power, lean body mass body fat, thigh muscle, thigh fat, and etc.
NCT03107884	Role of Metformin on Muscle Health of Older Adults	Muscle Atrophy, Insulin Resistance	Muscle size; Insulin sensitivity
NCT01765946	Metformin and Longevity Genes in Prediabetes	Insulin Resistance, Prediabetes, Aging, Inflammation	Longevity gene expression; Insulin sensitivity; Monocyte polarization status
NCT04375657	Thymus Regeneration, Immunorestoration, and Insulin Mitigation Extension Trial	Epigenetic Aging, Immunosenescence	Epigenetic age; Thymus regeneration; Safety and tolerability; Immunosenescence

## Abbreviations

AD: alzheimer's disease
AS: ankylosing spondylitis
ATM: ataxic telangiectasis mutation
circRNAs: circular RNAs
DPP4: dipeptidyl peptidase 4
FBG: fasting blood glucose
GRP78: glucose-regulated protein 78
GLP1: glucagon-like peptide 1
GPx7: glutathione peroxidase 7
HSP: heat shock protein
HGP: hepatic glucose production
HFD: high-fat-diet
H3K27me3: histone H3 lysine 27 trimethylation
HD: huntington's disease
IGF-1: insulin-like growth factor 1
IRS-1/2: insulin receptor substrate-1/2
IVD: intervertebral disc
IVDD: intervertebral disc degeneration
KLF2: krüppel-like factor 2
LVM: left ventricular mass
LVMI: left ventricular mass index
LKB1: liver kinase B1
LncRNA: long non-coding RNAs
NIDDM: non-insulin-dependent diabetes mellitus
Nrf2: nuclear factor erythroid 2-related factor 2
OCT: organic cation transporter
OA: osteoarthritis
OP: osteoporosis
OS: osteosarcoma
PDAC: pancreatic ductal adenocarcinoma
PD: parkinson's disease
PD: periodontitis
PCOS: polycystic ovarian syndrome
Rag: Ras-related GTP-binding protein
RA: rheumatoid arthritis
ROS: reactive oxygen species
SASP: senescence-associated secretory phenotype
SGLT2: sodium-dependent glucose transporters 2
TAME: targeting aging with metformin
TSC2: tuberous sclerosis complex 2
T2D: type 2 diabetes
VIS: virus-induced cellular senescence
